# Risk prediction model of gestational diabetes mellitus based on nomogram in a Chinese population cohort study

**DOI:** 10.1038/s41598-020-78164-x

**Published:** 2020-12-04

**Authors:** Xiaomei Zhang, Xin Zhao, Lili Huo, Ning Yuan, Jianbin Sun, Jing Du, Min Nan, Linong Ji

**Affiliations:** 1 Department of Endocrinology, Peking University International Hospital, Beijing, China; 2 Department of Endocrinology, Beijing Jishuitan Hospital, Beijing, China; 3 Department of Endocrinology, Peking University People’s Hospital, Beijing, 100001 China

**Keywords:** Endocrine system and metabolic diseases, Metabolic disorders

## Abstract

To build a risk prediction model of gestational diabetes mellitus using nomogram to provide a simple-to-use clinical basis for the early prediction of gestational diabetes mellitus (GDM). This study is a prospective cohort study including 1385 pregnant women. (1) It is showed that the risk of GDM in women aged ≥ 35 years was 5.5 times higher than that in women aged < 25 years (95% CI: 1.27–23.73, *p* < 0.05). In the first trimester, the risk of GDM in women with abnormal triglyceride who were in their first trimester was 2.1 times higher than that of lipid normal women (95% CI: 1.12–3.91, *p* < 0.05). The area under the ROC curve of the nomogram of was 0.728 (95% CI: 0.683–0.772), the sensitivity and specificity of the model were 0.716 and 0.652, respectively. This study provides a simple and economic nomogram for the early prediction of GDM risk in the first trimester, and it has certain accuracy.

## Introduction

Gestational diabetes mellitus (GDM) is abnormal glucose tolerance with onset or first recognition during pregnancy, which is one of the most common metabolic complications during pregnancy. In the recent years, with socio-economic development and improvement in living standards, diet and lifestyle have changed. In addition to these changes, the lack of understanding of the complications associated with pregnancy complications has increased the incidence of GDM in pregnant women. However, with the gradual improvement in the understanding of GDM, GDM screening has been established; this has improved the detection of GDM. GDM has short and long-term adverse effects on the health of the mother and offspring^1^. In addition to type 2 diabetes mellitus (T2DM), the long-term adverse effects of GDM on the mother also include cardiovascular disease and metabolic syndrome^2^. Furthermore, the short-term adverse effects of GDM include higher risk of pregnancy hypertension, preeclampsia, caesarean section, amniotic fluid excess, premature rupture of membranes, and ketoacidosis^3^. In addition, GDM increases the risk of neonatal complications such as birth injury, respiratory distress syndrome, hyperbilirubinemia, and hypoglycemia^4^. If the glucose level of pregnant women with GDM is not well controlled, it will lead to fetal hyperinsulinemia, neonatal hyperglycemia, and excessive growth of the fetal macrosomia^5^. The long-term adverse effects of GDM on offspring include the increased risk of T2DM and obesity^6,7^. Some studies have shown that Early diagnosis and treatment of GDM has been proved to be effective in improving pregnancy outcomes^8,9^. Because the current diagnosis of “OGTT” test of GDM is taken in the second trimester of pregnancy, which is in 24–28 weeks of gestation. Once the GDM was diagnosed, the insulin intervention combined with diet control would be needed. However, it may lose the opportunity of early intervention. Therefore, identifying the risk factors and establishing a simple, practical risk prediction model of GDM, especially in the first trimester, is of great clinical significance.

Many cross-sectional studies have reported the risk factors of GDM. Several mathematical models using the biological and clinical indicators have been developed to predict GDM. This study is to predict GDM with using the age, body mass index (BMI), and indicators of glycolipid metabolism, to enable early recognition and intervention of GDM.

## Results

### Comparison of general conditions and biochemical indices in the first trimester

Of the 1385 pregnant women 274 (19.78%) were diagnosed with GDM in the second trimester. The age of the participants in the GDM group (26.28%) significantly higher than those in the non-GDM group (χ^2^ = 20.54, *p* < 0.05). Compared with the non-GDM group, the proportion of multipara in the GDM group increased significantly (χ^2^ = 7.25, *p* < 0.05). In the first trimester, the BMI of GDM group was higher than that of the non-GDM group; the proportion of overweight and obese participants was higher in the GDM group compared with the non-GDM group (χ^2^ = 47.18, *p* < 0.05). The levels of HbA1c and FBG in the GDM group were significantly higher than those in the non-GDM group (*p* < 0.05). In addition, the level of TC, TG, LDL-C, and UA in GDM group were higher than those in non-GDM group (*p* < 0.05). SBP in the GDM group was higher than that in non-GDM group (*p* < 0.05), while there was no significant difference in DBP between the two groups. There was no significant difference on the level of SCr, Hcy, LPA, CRP, and NLR between the two groups (*p* > 0.05, respectively) (shown as Table 1).

**Table 1:** Comparison of general conditions and biochemical indexes in first trimester.

Index	Non-GDM group	GDM group	t (X^2^)	*p*
	(n = 1111)	(n = 274)		
**Age (years)**	30.69 ± 3.57	31.95 ± 3.79	− 5.15	< 0.05
< 25	27 (2.43%)	2 (0.73%)		
25–34	914 (82.27%)	200 (72.99%)		
≥ 35	170 (15.30%)	72 (26.28%)	20.54	< 0.05
**BMI (kg/m**^**2**^**)**	21.63 ± 2.81	23.09 ± 3.43	− 7.32	< 0.05
≤ 24	920 (82.81%)	179 (65.32%)		
24–28	160 (14.40%)	69 (25.18%)		
> 28	31 (2.79%)	26 (9.49%)	47.18	< 0.05
**Parity**	1.84 ± 0.93	2.09 ± 1.06	− 3.92	< 0.05
0	479 (43.11%)	93 (33.94%)		
> 1	632 (56.89%)	181 (66.06%)	7.25	< 0.05
SBP (mmHg)	109.58 ± 10.46	111.31 ± 10.51	− 2.44	< 0.05
DBP (mmHg)	66.18 ± 8.78	66.18 ± 8.91	− 0.01	0.98
TC (mmol/L)	3.91 ± 0.67	4.14 ± 0.79	− 5.01	< 0.05
TG (mmol/L)	0.96 ± 0.56	1.15 ± 0.80	− 4.48	< 0.05
LDL-C (mmol/L)	2.01 ± 0.53	2.16 ± 0.61	− 3.91	< 0.05
HDL-C (mmol/L)	1.41 ± 0.27	1.40 ± 0.63	0.39	0.14
UA (umol/L)	209.86 ± 47.41	227.56 ± 53.73	− 5.37	< 0.05
SCr (umol/L)	49.52 ± 8.27	48.68 ± 6.91	1.54	0.12
HbA1c (%)	5.07 ± 0.26	5.23 ± 0.30	− 6.91	< 0.05
FBG (mmol/L)	4.85 ± 0.35	5.01 ± 0.39	− 6.76	< 0.05
Hcy (umol/L)	6.61 ± 1.62	6.44 ± 1.48	1.57	0.12
CRP (mg/L)	2.26 ± 3.94	3.03 ± 3.78	− 0.40	0.38
Lpa (mg/L)	151.12 ± 179.57	156.12 ± 163.41	− 0.40	0.69
NLR	3.20 ± 1.03	3.26 ± 1.19	− 0.27	0.78

*BMI* body mass index, *SBP *systolic blood pressure, *DBP* diastolic blood pressure, *FBG* fasting blood glucose, *HbA1c* glycosylated hemoglobin, *SCr *serum creatinine, *UA* uric acid, *TC* total cholesterol, *TG* triglycerides, *LDL-C* low-density lipoprotein cholesterol, *HDL-C* high-density lipoprotein cholesterol, *Hcy* homocysteine, *CRP* C-reactive protein, *Lpa* lipoprotein-a, *NLR* neutrophil/lymphocyte ratio.

### Logistic regression analysis of risk factors of GDM

**Figure 1:**
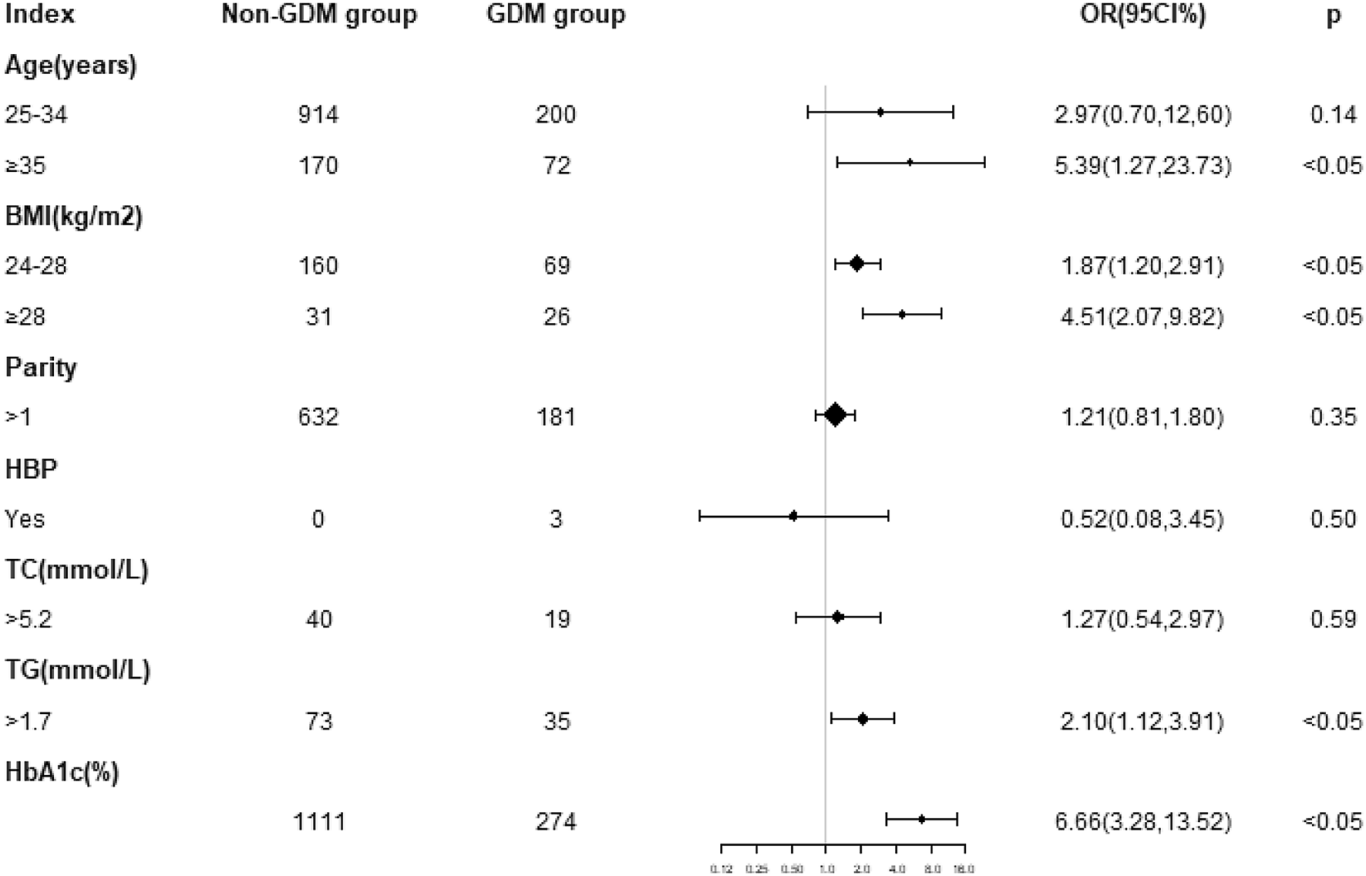
Forest map of GDM risk factors. BMI is for body mass index, HBP is for hypertension, HbA1c is for glycosylated hemoglobin, TC is for total cholesterol, TG is for triglycerides.

A multivariate logistic regression model was established with GDM as the dependent variable and single factor regression analysis as the independent variables, including age, BMI, parity, blood pressure, HbA1c, TC, and TG. The results showed that the risk of GDM in pregnant women aged ≥ 35 years was 5.5 times higher than that in pregnant women aged < 25 years (95% CI: 1.27–23.73, *p* < 0.05). The risk of GDM in women with BMI 24–28 kg/m^2^ in the first trimester was 1.9 times that in normal weight women (95% CI: 1.20–2.91, *p* < 0.05). The risk of GDM in women with BMI > 28 kg/m^2^ was 4.5 times that in women with normal weight (95% CI: 2.07–9.82, *p* < 0.05). The risk of GDM in women with higher TG was 2.1 times higher than that in women with normal TG (95% CI: 1.12–3.91, *p* < 0.05) (shown as Fig. 1).

### Nomogram and evaluation of prediction model of GDM

**Figure 2:**
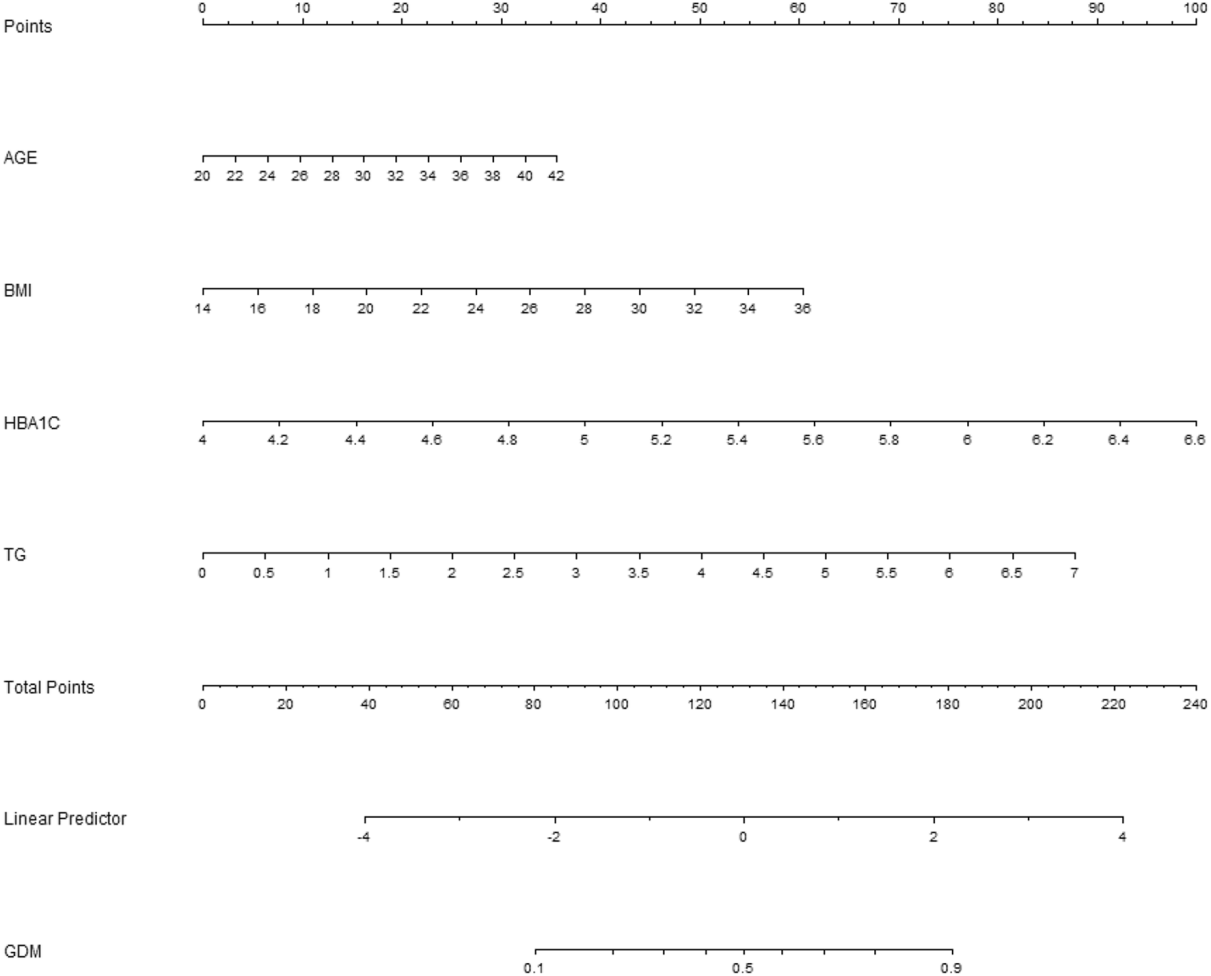
Nomogram for GDM in first trimester. Instructions: each individual’s GDM risk for patients was estimated by plotting on each variable axis. A vertical line was drawn from that value to the top points scale to determine the number of points that were assigned by that variable value. Then, the points from each variable value were summed. The sum on the total points scale was located and vertically projected onto the bottom axis, and then a personalized GDM risk was obtained. BMI is for body mass index, HBP is for hypertension, HbA1c is for glycosylated hemoglobin, TC is for total cholesterol, TG is for triglycerides.

**Figure 3:**
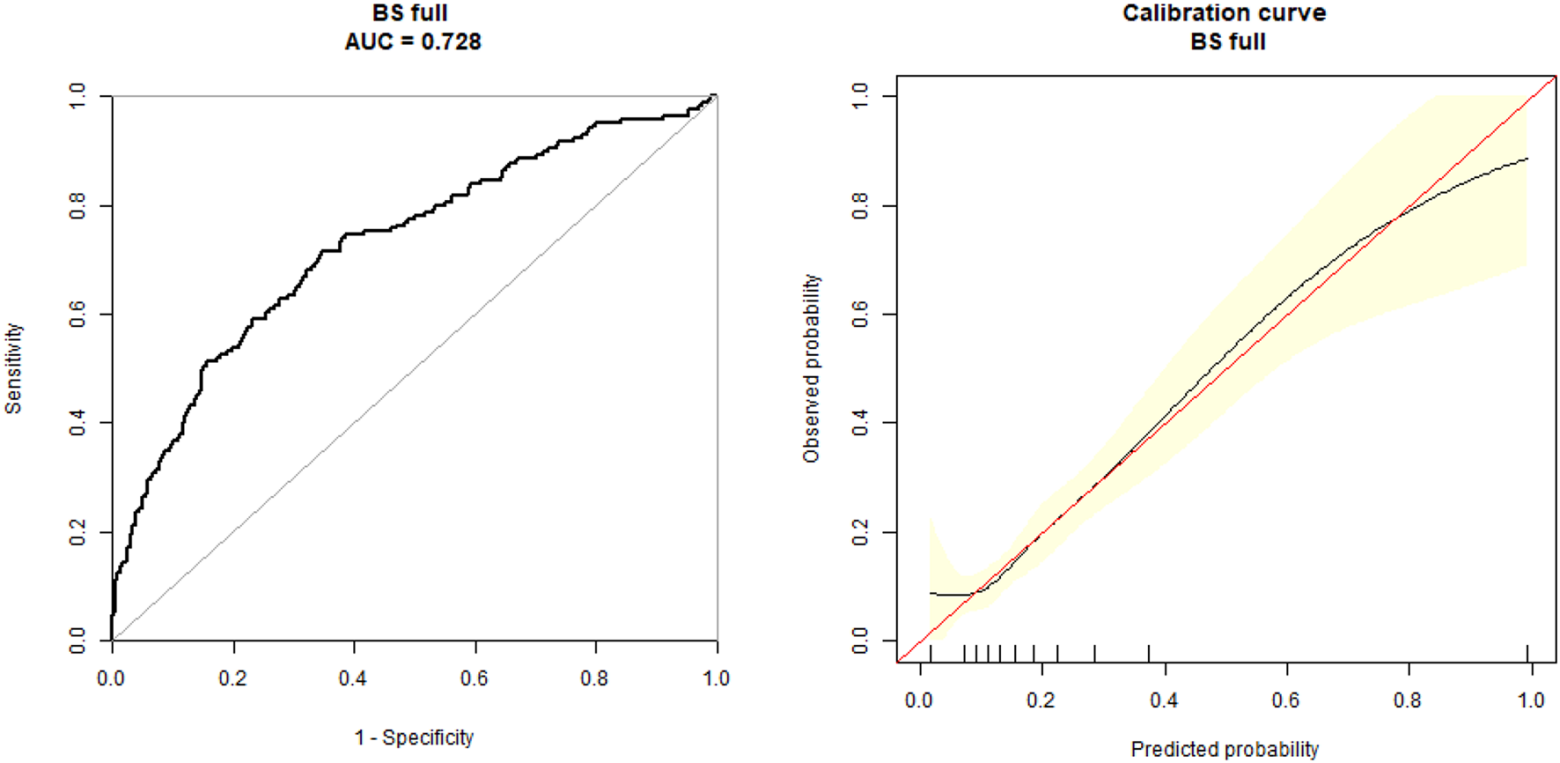
ROC curves for the accuracy of the GDM nomogram in patients. The overall predictive accuracy of the nomogram for GDM was 0.728 (95%CI: 0.683, 0.772), and the sensitivity and specificity were 0.716 and 0.652, respectively.

The nomogram of GDM risk prediction model of pregnant women was established based on the independent risk factors of GDM in the first trimester (shown as Fig. 2). The total score of GDM in the first trimester was calculated according to the corresponding score of each prediction factor to estimate the incidence probability of GDM. The AUC of GDM risk prediction model of GDM in the internal validation model population was 0.728 (95% CI: 0.683–0.772), which showed good discrimination ability. The sensitivity and specificity of the validation model was 0.716 and 0.652, respectively; the positive predictive value was 0.502, and the negative predictive value was 0.895 (shown as Fig. 3).

## Discussion

GDM is a common comprehensive disease syndrome in obstetrics and gynecology, which is closely related to the abnormal glucose metabolism during pregnancy. It is mainly related to the genetic factors of the patients themselves, the living and diet habits, and the daily work and rest habits^10^. In the women with GDM, there is no abnormal blood glucose metabolism in patients before pregnancy, and diagnosed as abnormal blood glucose after OGTT test usually performed at the 24–28th week of pregnancy. After the delivery, the blood glucose metabolism of most patients with GDM may return to normal, but some patients with GDM may develop T2DM. GDM can lead to perinatal complications, such as macrosomia, dystocia, caesarean section, and hypoglycemia. At the same time, GDM has long-term adverse effects on T2DM in the mother and causes obesity in the offspring. Early diagnosis and treatment of GDM have been proved effective in improving pregnancy outcome^11^.

Several T2DM risk prediction model^12–14^ and cardiovascular disease risk prediction model^15,16^ have been established. In recent years, with the increasing incidence rate of GDM, the number of studies on the prediction model of GDM risk is increasing. In the past decade, the number of predictive models developed in the field of obstetrics has been more than tripled^17^. Risk models are established by combining the patient’s general characteristics, clinical test results, genetic information, and other data^18^. Most of the GDM risk prediction models are based on age, nationality, and BMI before pregnancy; there is still a lack of other GDM related risk factors other than the basic characteristics of pregnant women as the prediction factors. Also, there is no intuitive and individualized risk model for predicting GDM. In addition, mostly studies been reported until now were cross-sectional retrospective. In this study, we conducted a longitudinal cohort study, and included more accurate clinical indicators observed in the first trimester to predict the risk of GDM.

We developed the risk prediction model of GDM based on the age of pregnant women, BMI in first trimester, parity, BP, blood lipid profile, UA, and inflammatory indices in the first trimester; thereafter, the final predictive factors of the model were determined by regression model. Finally, the nomogram was built and evaluated by verifying the model.

Recently, much attention has been paid to the role of inflammatory factors in the development of GDM. The prediction indices include TNFα^19^, interleukin^20^, leukocyte^21^, and high-sensitivity C-reactive protein (hsCRP)^22^. The prediction time includes the second and the first trimester of pregnancy, but there is no simple and practical prediction index with high sensitivity and specificity. Some studies showed that the level of NLR in pregnant women with GDM was significantly higher than that in pregnant women without GDM. However, some studies have contradicted the role of NLR in the prediction of GDM risk^21,23^. Berggren et al. conducted a prospective nested case–control study^24^ on women < 14 weeks pregnant. They showed that the hsCRP level of the pregnant women with abnormal glucose tolerance was higher than that of the pregnant women with normal blood glucose level; hsCRP was found to be positively correlated with the increase in blood glucose. However, correlation between hsCRP and GDM in the current study remains inconsistent^25^. In this study, the levels of CRP and NLR in the GDM group were higher than those in the non-GDM group, but the difference was not statistically significant (*p* > 0.05, respectively).

In pregnancy, in order to maintain the normal demand of pregnancy, the level of blood lipid will increase physiologically. To maintain a normal condition during pregnancy, the blood lipid level increases. Some studies have shown that in the first trimester the level of TG changes significantly, which may be related to the influence of progesterone and estrogen in the body, preferentially intake of high calorie food, to prepare for pregnancy^26^. However, with the improvement of living standard, change in diet and lifestyle, the increase of elder and multiple parity, the number of pregnant women with abnormal lipid metabolism is increasing. Although there are studies on the relationship between blood lipid metabolism and GDM, the results are not consistent^27^. Because GDM increases the rate of adverse pregnancy and childbirth, and affects the long-term health and life of the offspring, the early prediction of blood lipid level in GDM has become one of the hot spots of clinical research. Some studies have shown that pregnant women with GDM have increased TC, TG, and LDL-C, and decreased of HDL-C compared with pregnant women without GDM. However, the results of a meta-analysis showed that the changes of cholesterol is not consistent across different except for the increase of TG^28^. Many previous studies have shown that the abnormal metabolism of blood lipid is related to the occurrence of GDM due to the influence of age and BMI^29, 30^. The results of this study show that in the first trimester, the levels of TC, TG, and LDL-C in the GDM group were significantly higher than that in the non-GDM group; there was no significant difference in the levels of HDL and LPA between the two groups. Moreover, multivariate logistic regression showed that after adjusting age, BMI, and parity, the increase in TG in the first trimester was 2.1 times of the incidence of GDM compared with pregnant women with normal level of TG (95% CI: 1.12–3.91, *p* < 0.05). However, the increase of TC is not an independent risk factor for GDM. TG can be used as one of the indicators for GDM prediction. These results are not completely consistent with the previous studies.

According to the age of pregnant women, BMI in first trimester, and abnormal glucose and lipid metabolism, the risk prediction model of GDM was logit *P* = −15.53294 + 0.07077 * age + 0.11996 * BMI + 1.68155 * HbA1c + 0.54787 * TG. The AUC of nomogram for GDM risk prediction model was 0.728 (95% CI: 0.683–0.772) in the internal validation model, which showed good discrimination ability. The sensitivity, specificity, positive predictive value, and negative predictive value were 0.716, 0.652, 0.502, and 0.895, respectively.

In this study, blood lipid and HbA1c was used to predict GDM; this was simple, feasible, and practical. It can be carried out widely in primary and community health center. The combination of general conditions and multiple biochemical indicators in the first trimester can significantly improve the sensitivity and specificity of prediction; this could also help to predict and identify GDM, and promote early prevention, intervention, and treatment of GDM. Moreover, it can improve the pregnancy outcome, and reduce the incidence of long-term disease of mother and offspring. Finally, at present, in the field of study of GDM, most of the studies are about risk factors of GDM with the application of logistic regression and lack of prediction model. In this study, new statistical methods using R language was used to built the nomogram for predicting the GDM which may occuring in the second and third trimester during pregnancy. This model is more intuitive and individualized than those established based on the risk factors of GDM in pregnant woman. Meanwhile, our study is strictly in accordance with the correct prediction model construction and verification methods to evaluate the risk prediction model of GDM, in order to predict the risk of GDM in the second and third trimester during pregnancy.

There are some limitations of this study. The study was conducted in a single center and the sample size was small. In future studies, multi-center and a large sample size are needed to accommodate more patients for further study. In addition, this study is based on the cohort results of Chinese population, so it is only applied to the Chinese population, not to different ethnicities.

There were short-term and long-term threat on health of the mother and offspring due to GDM. In this study, it was found that age, BMI, TG, and HbA1c in the first trimester were the independent risk factors of GDM. These have certain predictive value for GDM, and the sensitivity and specificity of the combined prediction will be higher. Nomogram is a simple, fast, and economic method and it provides a new clinical method for early prediction and recognition of GDM.

## Materials and methods

### Participants

This study is a prospective cohort study. 1385 pregnant (gestational 7–12 weeks) women who were admitted to the obstetrics department of Peking University International Hospital from December 2017 to March 2019 were enrolled. Follow-up was performed regularly. Inclusion criteria were as follows: pregnant women aged > 18 years, have taken oral glucose tolerance test (OGTT) at 24–28 weeks of gestation; willing to undergo in-hospital birth examination and delivery; agreed to answer the relevant survey questionnaire, and provided blood samples.

Exclusion criteria were as follows: pregnant women with cardiovascular and cerebrovascular diseases, respiratory diseases, pre-pregnancy diabetes, thyroid diseases, hematological disorders, and liver and kidney diseases; multiple pregnancy women. This study was approved by the bioethics committee of Peking University International Hospital. All participants have signed the informed consent.

### Methods

#### General characteristics

(1) Basic information collected: the age, parity and gestational week of pregnant women were recorded during enrollment. (2) Height and weight measurement: height, and weight were measured; BMI was calculated as the formula: height (kg)/body weight^2^(m^2^). (3) Blood pressure including diastolic blood pressure (DBP) and systolic blood pressure (SBP) was measured in all participants.

#### Laboratory measurement

Venous blood samples were collected after at least 8 h of fasting in the morning. Laboratory measurements included routine blood test, neutrophil/lymphocyte ratio (NLR), glycosylated hemoglobin (HbA1c), fasting blood glucose (FBG), triglyceride (TG), total cholesterol (TC), high-density lipoprotein cholesterol (HDL-C), low-density lipoprotein cholesterol (LDL-C), creatinine (SCr), uric acid (UA), homocysteine (Hcy), lipoprotein-a (LPA), and C-reactive protein (CRP). HbA1c was measured using an HPLC with Dongcao G8 glycosylated hemoglobin analyzer.

#### GDM diagnosis

OGTT test was performed to screen GDM in 24–28 weeks of gestation. Pregnant women were admitted to hospital in the morning after 8–12 h of fasting. 75 g glucose powder was dissolved in 250–300 ml warm water and was taken orally within 5 min. The venous blood before, 1 h, and 2 h after were recorded.

IADPSG was used as the diagnostic standard of gestational diabetes^31^ was used: fasting blood glucose > 5.1 mmol/L, blood glucose at 1 h after glucose intake > 10.0 mmol/L, blood glucose at 2 h after glucose intake > 8.5 mmol/L was diagnosed as GDM.

### Statistical analysis

Data were analysed using the SPSS 21.0 software. Data with normal distributions were shown as mean ± standard ${{(\overline{{\text{x}}}}} \pm {\text{s)}}$ and non-normal were shown as median (interquartile range), respectively. We used T-test and Wilcoxon test to analyze the difference between GDM group and non-GDM group respectively. The χ^2^ test was used to compare the unit of count between the two groups. We use unconditional multivariate logistic regression analysis to calculate the odds ratios (ORs) and its 95% confidence intervals (95% CIs). The differences were considered statistically significant when *p* < 0.05.

The nomogram was performed with statistical packages R (http://www.R-project.org) and EmpowerStats (www.empowerstats.com, X&Y Solutions, Inc., Boston, MA). And the ROC curves and AUC were calculated to evaluate the accuracy, the sensitivity and the specificity of the model.

### Ethical approval

This study was approved by the bioethics committee of Peking University International Hospital. All procedures performed in studies involving human participants were in accordance with the ethical standards of the institutional and/or national research committee and with the 1964 Helsinki declaration and its later amendments or comparable ethical standards.

### Informed consent

Written informed consent was obtained from the individual participant included in the study.

## Data Availability

The datasets generated and analyzed during the current study are available from the corresponding author on reasonable request.
